# Impact of sensory organization tasks on prefrontal cortex activity in older women: a comparative fNIRS study of osteoarthritis and healthy aging

**DOI:** 10.3389/fnagi.2025.1583447

**Published:** 2025-06-13

**Authors:** Alka Bishnoi, Yang Hu, Manuel E. Hernandez

**Affiliations:** ^1^The College of Health Professions and Human Services, Department of Physical Therapy, Kean University, Union, NJ, United States; ^2^Department of Kinesiology, College of Health and Human Sciences, San Jose State University, San Jose, CA, United States; ^3^Carle Illinois College of Medicine, Department of Biomedical and Translational Sciences, University of Illinois Urbana-Champaign, Urbana, IL, United States; ^4^College of Applied Health Sciences, Department of Kinesiology and Community Health, University of Illinois Urbana-Champaign, Urbana, IL, United States

**Keywords:** near infrared spectroscopy, prefrontal cortex, osteoarthritis, sensory organization task, balance

## Abstract

**Introduction:**

Osteoarthritis (OA), a prevalent musculoskeletal condition, is associated with an increased risk of falls. Maintaining posture relies on visual, vestibular, and proprioceptive inputs, but these systems can be compromised due to aging or disease, heightening fall risk. Such impairments may result from neuromuscular decline and reduced cognitive or visuospatial processing abilities. This study aimed to investigate prefrontal cortical (PFC) activation patterns during clinical sensory organization tasks (SOT) using functional near-infrared spectroscopy (fNIRS) in older women with OA and healthy controls (HOA). We hypothesized that PFC activation would increase as SOT conditions became more challenging, but that increases would be limited in OA, relative to HOA, given a decreased attentional capacity due to chronic pain.

**Methods:**

A cross-sectional study was conducted with 10 women with OA (65.7 ± 3.01 years) and 11 HOA (66.0 ± 4.86 years). Baseline cognitive and motor assessments preceded three trials of six SOT conditions.

**Results:**

Significant differences between groups in BMI, WOMAC pain score, repeated chair stand, and TUG scores were found (*p* < 0.001). Linear mixed-model analysis revealed significant effects of condition (CND; *p* < 0.001), trial (TR; *p* < 0.0001), and interactions between CND^*^TR (*p* < 0.01) and Cohort^*^CND (*p* < 0.01) on PFC activation.

**Discussion:**

In conclusion, both groups demonstrated increased PFC activation with task difficulty. However, OA participants exhibited diminished capacity to recruit additional attentional resources compared to HOA, emphasizing the need for further research with larger cohorts to elucidate these findings.

## 1 Introduction

Osteoarthritis (OA) affects ~32.5 million adults in the United States (Centers for Disease Control and Prevention, 2020). OA is a degenerative joint disease impacting articular cartilage (Loeser, 2017), and it is particularly prevalent among women over the age of 50, especially those of Caucasian or African American descent (Vaughn et al., 2019). The risk factors for OA include gender, obesity, and race (Centers for Disease Control and Prevention, 2020), with aging being a primary contributor (Loeser, 2017). Aging not only elevates the likelihood of developing OA but also heightens fall risk, with about 30%−40% of older adults experiencing falls each year (Loeser, 2017).

Maintaining mobility, particularly balance, is a dynamic process that relies on input from the central nervous system (CNS) (Faraldo-García et al., 2016). CNS integrates information from multiple sources to maintain stability and function, highlighting the importance of a stable field of vision, a well-functioning vestibulo-ocular system, and adequate muscle strength (Wittenberg et al., 2017). Age-related declines in posture and responsiveness to external stimuli can increase fall risk and impair mobility, which are associated with greater morbidity and mortality in older adults (Faraldo-García et al., 2012, 2016).

As the CNS undergoes age-related changes, balance and gait are often compromised (Faraldo-García et al., 2012). The frontal lobe plays a crucial role in executive functions and decision-making, which are also essential for gait and balance control (Coppin et al., 2006). The prefrontal cortex (PFC), in particular, is instrumental in motor actions (Mihara et al., 2008), suggesting that monitoring PFC activation in postural control tasks may provide valuable insights, given the role of the PFC in executive function, decision-making, and possibly compensatory mechanisms in balance control. Balance is integral to daily activities, such as maintaining posture and reacting to external stimuli, including unexpected perturbations during walking (Bishnoi et al., 2024). Studies indicate that pain may contribute to declines in executive function and an increased prevalence of balance disturbances in older adults (van der Leeuw et al., 2016; Murata et al., 2017). Lastly, greater pain severity has been linked to impaired executive function in community-dwelling older individuals (Murata et al., 2017).

In OA patients, joint pain and stiffness—key symptoms of the disease—may compromise postural control, further impacting the executive functions required for daily activities. Painful stimulation had resulted in increased activation in anterior cingulate cortex and right dorsolateral PFC in hip OA patients (Gwilym et al., 2009). If left unassessed, joint pain and stiffness could exacerbate cognitive decline, increase healthcare costs, and lead to higher rates of hospitalization. Recent studies showed that patients with chronic musculoskeletal pain exhibited increased neural responses to painful stimuli in different parts of PFC (Derbyshire et al., 2002; Gracely et al., 2002; Üçeyler et al., 2015). However, further examination of brain activity during postural control tasks is needed, as exercise has been found to be associated with decreased PFC activation in OA (Öztürk et al., 2021). Recent advancements, such as Functional Near-Infrared Spectroscopy (fNIRS), allow for the measurement of PFC activation by capturing hemodynamic responses during specific tasks (Holtzer et al., 2014), and has demonstrated decreased PFC activation during perturbation walking tasks in OA, relative to HOA (Bishnoi et al., 2024).

The objective of this study was to investigate changes in PFC activation during the Sensory Organization Task (SOT) in older adults with and without OA. We utilized fNIRS to assess fluctuations in oxyhemoglobin (HbO_2_) and deoxyhemoglobin (Hb) levels during SOT tasks, aiming to clarify the brain's role in balance maintenance in this population. We hypothesized that PFC activation would increase as SOT conditions became more challenging, but that the increase would be limited in OA, relative to HOA, given a decreased attentional capacity due to chronic pain. This hypothesis is informed by recent work in OA while walking with perturbations (Bishnoi et al., 2024), and the sensory organization task is likely associated with both balance control and cognitive-motor integration. For secondary analysis, we examined the balance performance during sensory organization tasks. We hypothesized that adults with OA would show decreases in balance performance during the SOT in comparison to adults without OA. Secondarily, we examined regional PFC activity among the six conditions in the two cohorts and examined correlations between PFC activation levels and independent variables (Time up and go test (TUG), WOMAC pain score, body mass index (BMI), repeated chair stand (RCS) test, and equilibrium scores).

## 2 Material and methods

### 2.1 Participants

Ten older women with Osteoarthritis (65.7 ± 3.01 years of age) and 11 without osteoarthritis (66 ± 4.86 years of age) participated in this study. They were recruited from the local community for this two-session cross-sectional study. Inclusion criteria consisted of symptomatic OA of mild to high severity in older women. Exclusion criteria consisted of the presence of any neurological disorder and/or musculoskeletal condition aside from OA. All participants signed a written consent form approved by the local institutional review board prior to testing. Based on prior work examining the differences in hemodynamics during painful stimuli in older adults with osteoarthritis (Öztürk et al., 2021), a sample size of eight OA and eight HOA participants was needed to detect a significant effect at the *p* = 0.05 level with 0.90 power and an effect size *f* = 0.8, based on an *a priori* sample size analysis using G^*^Power (Version 3.1.9.6).

### 2.2 Protocol

**Figure 1:**
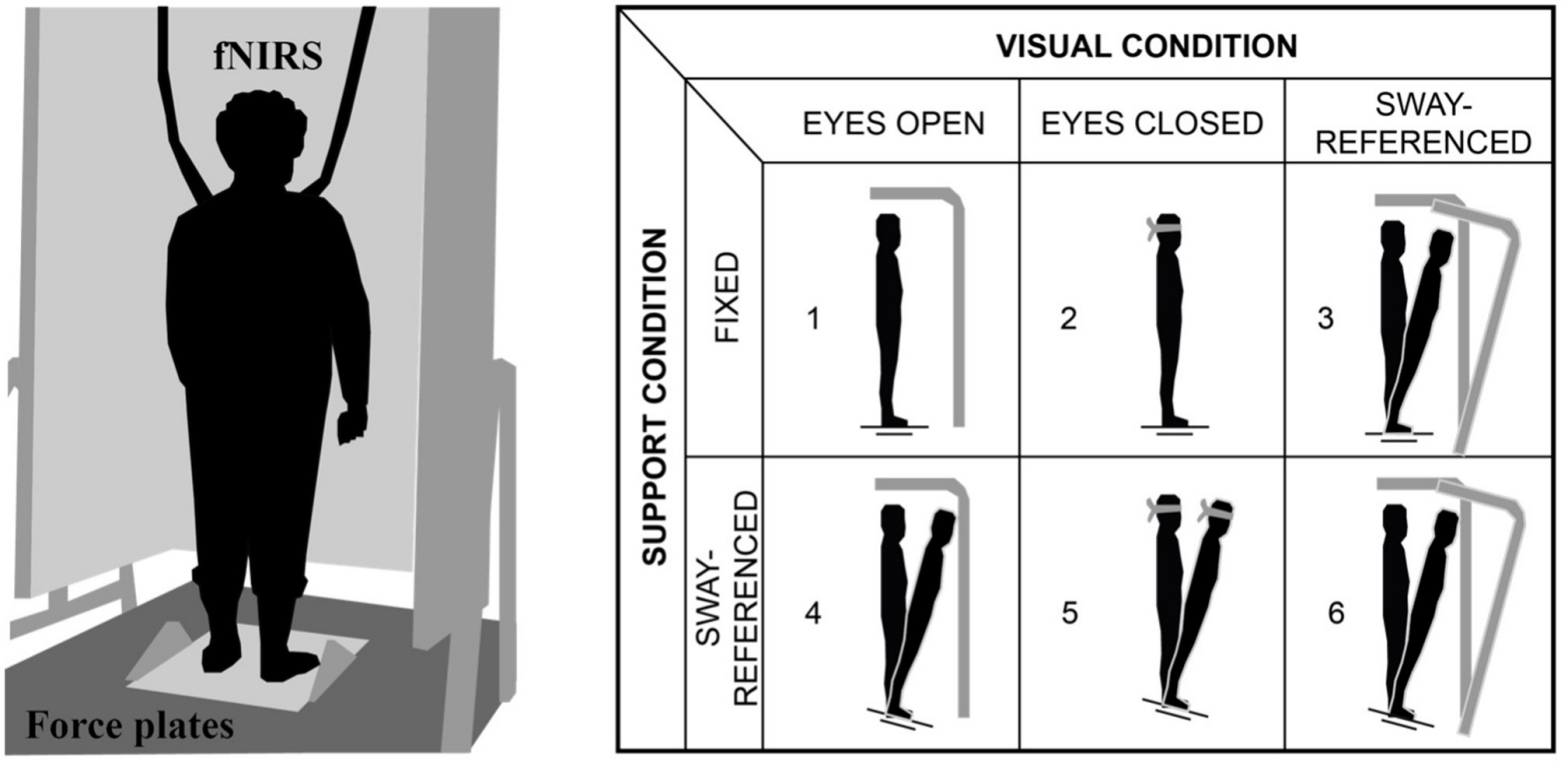
**(Left)** Experimental set up and **(Right)** sensory organization task (SOT) conditions.

Each participant completed 2-day testing in the Mobility and Fall Prevention Research Laboratory. Day 1 consisted of cognitive assessment including Repeated Battery for the Assessment of Neuropsychological Status (RBANS), and motor behavioral assessment consisted of Mini-Balance Evaluation Systems Test (Mini-BEST). Participants performed the Clinical Sensory Organization test on Neurocom while wearing FNIRS device. This test consisted of six conditions including three trials of 20 s each with a 30 s inter-trial break (Teo et al., 2018): condition 1: Eyes open (EO), condition 2: Eyes closed (EYC), condition 3: Eyes open sway reference moving (EYO_SR), condition 4: Eyes open plate reference moving (EYO_PR), condition 5: Eyes closed plate reference moving (EYC_PR), and condition 6: Eyes open sway and plate reference moving (EYO_SPR) (Teo et al., 2018) (Figure 1).

### 2.3 Physical and cognitive assessment

During their first visit, participants had their cognitive function assessed using the Repeated Battery for the Assessment of Neuropsychological Status (RBANS) (Randolph et al., 1998). For self-reported pain questionnaires, Western Ontario and McMaster Universities Arthritis Index (WOMAC) assessed the individuals' pain scale and activity (Salaffi et al., 2003) (Table 1). Physical assessments included the Gait Speed (GS) examined on instrumented treadmill and Mini-Balance evaluation systems test (Mini-BEST) (Madhavan and Bishnoi, 2017).

**Table 1:** Descriptive characteristics of participants.

**Characteristics**	**OA (*n* = 10)**	**HOA (*n* = 11)**	***p*-value**
Age (years)	65.7 ± 3.01	66 ± 4.86	0.80
BMI (kg/m^2^)	27.96 ± 5.42	22.61 ± 2.85	0.044^**^
WS-Pain (0–20)	4.30 ± 3.61	0.45 ± 0.65	0.002^**^
TUG-ST (seconds)	11.79 ± 1.37	9.94 ± 1.30	0.007^***^
TUG-DT (seconds)	13.67 ± 2.81	11.23 ± 1.78	0.04^*^
RCS (seconds)	13.27 ± 3.99	10.09 ± 2.91	0.03^*^

^**^p = 0.01, ^*^p = 0.05, ^***^p = 0.001.

OA, osteoarthritis; HOA, healthy older adults; WS, WOMAC score; TUG, time up and go test in Mini-BEST; ST, single task; RCS, repeated chair stand in mini-BEST.

### 2.4 Functional near infrared spectroscopy (FNIRS) data acquisition

FNIRS data was obtained using an fNIRS Imager 1200 system (fNIRS Devices, LLC, Potomac, MD). The headband sensor contained 10 photodetectors and 4 LED light sources with a 2.5 cm source detector separation distance that covered the forehead with the use of 16 optodes and a 2 Hz sampling rate. The light sources on the sensor (Epitex Inc. type L6X730/6X850) contain two built-in LEDs having peak wavelengths at 730 and 850 nm, with an overall outer diameter of 9.2 ± 0.2 mm. The photodetectors (Bur Brown, type OPT101) are monolithic photodiodes with a single supply transimpedance amplifier. The center of the headband sensor was placed on the central point of the forehead above the nasion (Fpz) in accordance with the 10/20 electroencephalography system, so that FP1 and FP2 marker locations were positioned on the bottom row of optodes and wide coverage of the PFC (e.g., orbitofrontal, ventrolateral, and dorsolateral PFC) was provided. Relative HbO_2_ and Hb levels (μM) were used because of their reliability in evaluating cortical activation changes (Doi et al., 2013).

The fNIRS data were collected using COBI Studio software and processed and analyzed using custom MATLAB scripts. Visual inspection of raw data was used to monitor for excessive noise, saturation, or dark current conditions. Using raw intensity measurements, ambient light levels below 100 μM, and 730 and 850 nm wavelength levels between 200–2,000 μM were confirmed in each optode included for analysis. To minimize the effects of physiological artifacts (e.g., breathing and heart rate) and any additional noise, the raw data were filtered using a low-pass filter with a cut-off frequency at 0.14 Hz (Izzetoglu et al., 2010). HbO_2_ and Hb levels (μM) were then calculated using the modified *Beer-Lambert law* for each of the 16 channels (Strangman et al., 2002).

PFC activation levels were assessed during the six SOT conditions with three trials of each condition. Each condition started with 10 s of standing quietly. All participants were asked to look forward and count silently staring from 1 in their head during this 10 s period. After that 10 s period, the instructions on the specific condition were provided. The 10 s baseline before each condition was used as a reference for both HbO_2_ and Hb relative levels (μM) (Ohsugi et al., 2013). To minimize the effects of fatigue, participants were given the opportunity of rest breaks between the conditions.

### 2.5 Statistical analysis

Custom MATLAB scripts were used for exporting to R, version 3.1.1. The Shapiro-Wilk test was done to check if data was normally distributed. All normally distributed data was used to do further analysis to explore the correlation between variables of interest. If the data was not normally distributed, we used nonparametric tests to check significance between variables of interest. Statistical significance was set at 0.05.

**Figure 2:**
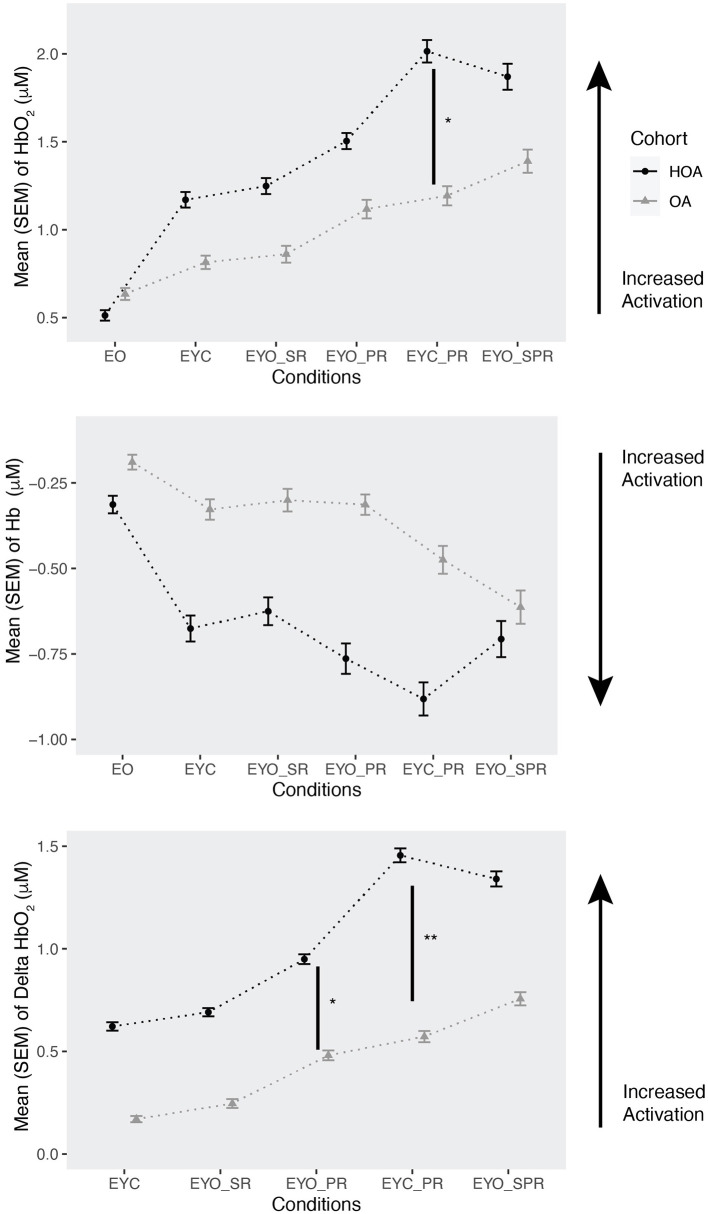
Mean (SEM) of oxyhemoglobin (HbO_2_) (μM), deoxyhemoglobin (Hb) levels (μM) and delta HbO2 levels in adults with (OA) and without osteoarthritis (HOA). Conditions are represented with increase in difficulty level: EO (eyes open), EYC (eyes closed), EYO_SR (eyes open with sway reference), EYO_PR (eyes open with plate reference), EYC_PR (eyes closed with plate reference), EYO_SPR (eyes open with sway and plate reference). HbO_2_ and Hb levels are reported as per the order of the conditions. Arrow indicates increase in neural activation and asterisks indicate *post hoc* results. *p = 0.05, **p = 0.01.

To test the primary hypothesis, linear mixed model analysis was used to examine PFC activation differences between groups (HOA and OA), conditions (1–6), and trials (1, 2, and 3). Data was visually inspected and the assumptions for the linear mixed model test were met. To control multiple comparisons, *post-hoc t*-tests were carried out using lsmeans Tukey's method in R and are denoted by asterisks in Figure 2. Secondary analysis included delta PFC [difference between HbO_2_ measure (μM) of conditions (2–6) and HbO_2_ measure (μM) of condition 1] and regional PFC analysis among the six conditions in the two cohorts.

Also, for exploration analysis, we examined the correlation between PFC activation levels and independent variables (TUG, WOMAC pain score, BMI, RCS, and Equilibrium score condition 3). Additionally, we analyzed variations in balance performance during sensory organization tasks.

## 3 Results

### 3.1 Participants

Participant characteristics are presented in Table 1. As expected, there were significant group differences among BMI, WOMAC, Repeated chair stand (RCS), Time Up and go Test-single task (TUG-ST), and TUG-Dual task (TUG-DT). BMI was significantly higher for the OA group than the HOA group, based on which, their pain scores and RCS time might be higher, which affected their time up and go test time as well during single task and dual task (Table 1).

### 3.2 PFC activation

Overall, PFC activation increased as task difficulty increased from EO to EYO_SPR (Table 2). This includes an increase in mean oxyhemoglobin (HbO_2_) levels and decrease in mean deoxyhemoglobin (Hb) levels. The most difficult condition among both cohorts was EYC_PR, followed by EYO_SPR, EYO_PR, EYO_SR, and lastly EYC. HbO_2_ levels in both OA and HOA increased as task difficulty increased. However, PFC activation was lower for OA population compared to HOA as task difficulty increased. In addition, there was significant interaction between OA cohort and in conditions (3, 4, 5, 6) in HbO_2_ levels, with OA demonstrating a decrease in HbO_2_ levels as difficulty of task increased in comparison to HOA. In Hb levels, there was significant difference between conditions, with EYC_PR showing a decrease in Hb levels as compared to easier tasks, suggesting an increase in PFC activation.

**Table 2:** Effects of conditions on HbO_2_ (μM), Hb levels (μM), and Delta HbO_2_.

**Fixed effects: condition**	**Estimate**	**SE**	***p*-value**
**Mean oxyhemoglobin (HbO**_2_ **) linear mixed model main effects and interactions**			
EYC	0.63	0.19	0.001^***^
EYO_SR	0.70	0.19	0.0005^***^
EYO_PR	0.97	0.19	<0.0001^***^
EYO_SPR	1.39	0.19	<0.0001^***^
EYC_PR	1.49	0.19	<0.0001^***^
**Cohort** ^*^ **condition**			
OA^*^EYC_PR	−1.04	0.29	0.0005^***^
OA^*^EYO_PR	−0.67	0.29	0.02^*^
OA^*^EYO_SPR	−0.65	0.29	0.03^*^
OA^*^EYO_SR	−0.59	0.29	0.05^**^
**Mean deoxyhemoglobin (Hb) linear mixed model main effects**			
EYC_PR	−0.53	0.12	<0.0001^***^
EYO_PR	−0.42	0.12	0.0008^***^
EYO_SPR	−0.37	0.12	0.003^***^
EYC	−0.35	0.12	0.005^***^
EYO_SR	−0.27	0.12	0.03^*^
**Delta PFC (HbO**_2_ **) linear mixed model main effects**			
EYC_PR	0.71	0.16	<0.0001^***^
EYO_PR	0.32	0.16	0.04^*^
EYO_SPR	0.61	0.16	0.0003^***^

Significance set at ^*^p < 0.05, ^**^p < 0.01, ^***^p < 0.001.

SE, standard error; HbO_2_, oxygenated hemoglobin; Hb, deoxyhemoglobin; Delta HbO_2_, change in HbO_2_ during easier task (condition 1) and difficult task (condition 2–6).

Conditions: 1: EO (eyes open), 2: EYC (eyes closed), 3: EYO_SR (eyes open with sway reference), 4: EYO_PR (eyes open with plate reference), 5: EYC_PR (eyes closed with plate reference), 6: EYO_SPR (eyes open with sway and plate reference).

For our secondary analysis, we evaluated the effect of conditions on Delta PFC among both cohorts (Table 2). Delta PFC was calculated through the difference between HbO_2_ measure (μM) of conditions (2–6) and HbO_2_ measure (μM) of condition 1. The results showed condition main effects only. Through delta PFC, we found higher activation in both groups during difficult conditions 4, 5, 6 (EYO_PR, EYC_PR, EYO_SPR). In regional PFC analysis, we did not find any significant group effects with HbO_2_ (Hemisphere: β = −0.03, SE = 0.12, *p* = 0.77; Region: β = −0.23, SE = 0.30, *p* = 0.45) or Hb levels (Hemisphere: β = 0.12, SE = 0.15, *p* = 0.45; Region: β = 0.14, SE = 0.18, *p* = 0.46), and nor the interaction effects with groups in HbO_2_ (Hemisphere: β = −0.05, SE = 0.17, *p* = 0.76; Region: β = −0.18, SE = 0.45, *p* = 0.68) or Hb (Hemisphere: β = −0.27, SE = 0.23, *p* = 0.29; Region: β = 0.21, SE = 0.27, *p* = 0.46) levels.

### 3.3 Clinical sensory organization task performance

Overall, there were no significant differences in composite scores between healthy older adults (HOA) and those with osteoarthritis (OA) (*p* > 0.05). However, a significant difference in equilibrium scores emerged during condition 3, eyes open with sway reference (EYO_SR; *p* = 0.02). Specifically, adults with OA demonstrated a lower equilibrium score compared to HOA. These findings suggest that individuals with OA face greater difficulty maintaining their center of gravity within a defined stability limit, particularly under the eyes open with sway reference condition, relative to other conditions.

### 3.4 Correlations

We did not find any significant correlations between prefrontal cortex (PFC) activation measures and Timed Up and Go (TUG) performance, Western Ontario and McMaster Universities Osteoarthritis Index (WOMAC) pain scores, or body mass index (BMI) in adults with osteoarthritis (OA; *p* > 0.05). Also, we didn't find any significant relationship between PFC activation variables and equilibrium balance score of condition in adults with and without OA.

In contrast, significant correlations emerged in healthy older adults (HOA) between deoxyhemoglobin (Hb) levels and TUG performance under specific conditions. During the eyes open plate reference moving (EYO_PR) condition, deoxyhemoglobin (Hb) was significantly associated with TUG single-task performance (Pearson's *r* = 0.63, *p* = 0.03). Similarly, during the eyes closed plate reference moving (EYC_PR) condition, significant associations were observed between deoxyhemoglobin (Hb) and TUG single-task performance (Pearson's *r* = 0.74, *p* = 0.009) as well as TUG dual-task performance (Pearson's *r* = 0.63, *p* = 0.04). No additional significant correlations were identified in either group.

## 4 Discussion

This study represents the first study to examine the relationship between PFC activation and balance control in older adults with and without osteoarthritis, to the authors' knowledge. Using fNIRS, we supported our hypothesis that the OA group would show a smaller increase in PFC activation as tasks progressed from easier to more challenging, relative to HOA group, consistent with lower attentional resources in OA. Further, our findings partly supported our hypothesis that the OA group would show decreases in balance performance during the SOT in comparison to HOA, consistent with prior postural control changes due to OA in more balance-demanding tasks.

### 4.1 Primary findings of the study

One of the most notable findings in our study was the observed difference in attentional resource recruitment between the OA and HOA groups. Specifically, the OA group exhibited difficulty mobilizing additional cognitive resources to meet task demands, a limitation that the HOA group did not display. As task difficulty increased, the cognitive demand appeared to surpass the resources available to the OA group, resulting in task disengagement and a reduction in HbO_2_ levels (Kahya et al., 2019). This contrast in activation patterns between the two groups suggests that OA may impair the ability to dynamically allocate attention and cognitive resources in response to challenging motor tasks. Alternatively, there may be an exercise-induced reduction of cortical activity seen in OA, but not HOA, which merits further exploration at rest, or before and after exercise, as in prior work (Öztürk et al., 2021), or reduced attentional resources in OA due to pain. In the HOA group, PFC activation increased with task complexity, marked by rising HbO_2_ and delta PFC levels, and decreasing Hb levels, reflecting efficient recruitment of attentional resources despite the increasing demands. These findings align with those of Herold et al. (2017), who also reported increased HbO_2_ levels during more complex balance tasks, supporting the hypothesis that greater neural activation corresponds to higher cognitive demand (Herold et al., 2017).

The initial expectation was that both groups would show increased PFC activation with task difficulty, given the heightened cognitive load associated with balance control in older adults. As the first study to assess PFC activation during sensory organization tasks (SOT) in adults with OA, we approached the results with a degree of uncertainty, particularly regarding the comparative responses between the OA and HOA groups. Our hypothesis was that the OA group would show a more significant increase in PFC activation than the HOA group as task complexity intensified, due to the expected challenge in balance compensation for OA-related impairments. However, OA participants instead recruited fewer attentional resources at the highest task difficulty level compared to HOA participants. This unexpected result suggests that individuals with OA might engage alternative cortical regions, perhaps due to limitations in the PFC's capacity to meet task demands under challenging conditions (Kahya et al., 2019).

### 4.2 Deoxyhemoglobin changes during SOT

In addition to changes in HbO_2_, our study identified significant reductions in Hb across conditions, which reinforces the observed inverse relationship between Hb and HbO_2_ levels in response to increasing task difficulty. This pattern implies a rising demand for oxygen and blood flow in the PFC, a trend that is consistent with previous studies reporting a decrease in Hb as task complexity rises, as seen in Teo et al. (2018). The observed increases in HbO_2_ levels and decreases in Hb levels collectively point to heightened activation in the PFC during balance challenges, particularly in HOA adults. These findings underscore the critical role of the PFC in compensatory neural responses to balance perturbations and highlight the potential limitations in cognitive resource allocation in adults with OA.

### 4.3 Age and disease related effects on cortical activation

This study also highlights how aging and disease-related changes in balance and proprioceptive function may influence cortical activation patterns. Age-related deterioration in proprioceptive feedback and the integrity of the neuromuscular system can compromise postural control, which may, in turn, increase reliance on higher cognitive resources, such as those provided by the PFC, for balance maintenance (Faraldo-García et al., 2016). The heightened cortical activation observed in the HOA group suggests effective recruitment of these resources; however, the relatively reduced recruitment in the OA group indicates potential deficits in compensatory responses due to OA-related impairments. While pain is often associated with executive function decline (Murata et al., 2017), suggestive of neural inefficiency, prior work has also indicated increased PFC activation in chronic pain (Gwilym et al., 2009), which would suggest a pain-related cognitive load. While we found no significant correlation between WOMAC pain scores and PFC activation levels in this study, the interactions observed between cohort and condition on PFC activity are consistent with decreased inefficiency in OA. However, the causality underlying neural inefficiency remains unclear, as inflammation may play a role in neuroinflammation and changes in neuronal activity (Vezzani and Viviani, 2015; Matsuda et al., 2019). Given that compensatory shifts to other interconnected regions, such as the parietal cortex, may be possible, the limited sample size and coverage of only the PFC constrains our ability to fully explore pain's impact on cortical responses, leaving this as an area for future investigation. Nevertheless, our findings align with previous literature suggesting that postural tasks lead to increased activation in cortical regions responsible for attentional and executive control (Karim et al., 2013).

### 4.4 Relationship between balance performance and osteoarthritis

Our secondary hypothesis aimed to explore differences in balance performance between OA and HOA during SOT. We anticipated that individuals with OA would exhibit decreased balance performance, particularly during the more challenging SOT conditions. Contrary to our expectations, we found only one task to be significantly different in balance performance: during the eyes open with sway reference, individuals with OA demonstrated reduced equilibrium score compared to the HOA group.

This result may indicate a heightened dependency on visual input among adults with OA, potentially leading them to be at increase fall risk when visual feedback is unavailable. A visual dependency in OA may reflect an adaptive strategy to overcome chronic sensory deficits. In contrast, HOA participants appeared to be more stable, which may suggest different compensatory mechanisms between the groups when visual input is removed. This finding highlights the potential adaptations in sensory reliance and balance strategy in OA and underscores the role of visual dependency in this population.

### 4.5 Limitations of the study

Despite the important insights gained from this study, several limitations warrant consideration. Firstly, our small sample size limits the generalizability of these findings and affected the correlations between variables which did not yield significant results, restricting us to explore further relationships, suggesting the need for replication in larger cohorts to confirm the observed trends. Secondly, our sample included participants with various OA types (e.g., hip, knee, spine), potentially affecting the generalizability of our results. Each OA type may impose different demands on postural control and may variably influence pain, cognitive function, and cortical activation. Future studies should consider stratifying participants by OA type to more precisely determine the effect of OA on cortical activation during balance tasks, as the severity and type of OA could differently impact executive functioning and balance in older adults. In addition, exploring other cortical regions beyond the PFC might provide further insight into the neural adaptations employed by adults with OA. Previous studies have suggested that older adults may recruit additional brain regions, such as the dorsolateral prefrontal cortex or motor cortex, to compensate for cognitive and motor demands. Understanding how OA affects these compensatory mechanisms could reveal potential intervention targets for improving balance and reducing fall risk in this population. Furthermore, a longitudinal approach could help clarify the progression of cortical changes in OA and their relationship to functional decline, offering insight into early detection and intervention strategies aimed at enhancing quality of life and independence in older adults with OA.

## 5 Conclusion

This study provides novel insights into the neural mechanisms underlying balance control in adults with osteoarthritis (OA), suggesting that OA may reduce the capacity to recruit additional cognitive resources when faced with challenging balance tasks. Specifically, the results indicate that adults with OA may struggle to increase prefrontal cortical (PFC) activation as task difficulty escalates, which could reflect limitations in their ability to adaptively engage cognitive resources necessary for postural stability under complex conditions. This impaired recruitment could be due to heightened reliance on visual inputs and primary motor strategies, potentially leaving less cognitive reserve available for dynamic postural adjustments.

The findings underscore the need for comprehensive interventions that simultaneously address cognitive and motor deficits in OA. Traditional balance training, effective in improving performance in OA (UzunkulaoGlu et al., 2020; Pirayeh et al., 2022), may benefit from incorporating cognitive elements, such as dual-task training or exercises designed to enhance attentional flexibility and executive function, which are critical for adapting to environmental demands. Cognitive-motor interventions, which integrate serial 3 subtraction, or interventions aimed at improving (Hamacher et al., 2016; Levinger et al., 2016; Genç and Demircioğlu, 2024) multi-sensory integration particularly through the use of virtual reality (Gomiero et al., 2018; Kuş et al., 2023) could help OA patients better manage postural challenges and improve overall stability and pain, especially in environments requiring rapid adaptation to sensory changes (Elshazly et al., 2016; Sarkar et al., 2022).

Furthermore, these insights into neural activation patterns open potential for using neuroimaging tools like functional near-infrared spectroscopy (fNIRS) to monitor and individualize treatment progress in adults with OA. Fall prevention programs could be tailored to target specific deficits in sensory integration and attentional control, particularly for those with advanced OA who may be at higher risk for falls and mobility impairments. This approach not only has implications for improving mobility but may also reduce healthcare costs associated with OA by mitigating risks for falls and subsequent injuries. Addressing both cognitive and motor components through targeted rehabilitation could thus foster improved quality of life and functional independence in this growing population.

## Author's note

The results of this manuscript were partly presented in the Society of Neuroscience Conference, 2020.

## Data Availability

The raw data supporting the conclusions of this article will be made available by the authors, without undue reservation.
